# New insights into the inter-organ crosstalk mediated by ChREBP

**DOI:** 10.3389/fendo.2023.1095440

**Published:** 2023-02-27

**Authors:** Thais Carbinatti, Marion Régnier, Lucia Parlati, Fadila Benhamed, Catherine Postic

**Affiliations:** Université Paris Cité, Institut Cochin, CNRS, INSERM, Paris, France

**Keywords:** ChREBP, inter-organ crosstalk, metabolism, hepatokines, metabolic diseases

## Abstract

Carbohydrate response element binding protein (ChREBP) is a glucose responsive transcription factor recognized by its critical role in the transcriptional control of glycolysis and *de novo* lipogenesis. Substantial advances in the field have revealed novel ChREBP functions. Indeed, due to its actions in different tissues, ChREBP modulates the inter-organ communication through secretion of peptides and lipid factors, ensuring metabolic homeostasis. Dysregulation of these orchestrated interactions is associated with development of metabolic diseases such as type 2 diabetes (T2D) and non-alcoholic fatty liver disease (NAFLD). Here, we recapitulate the current knowledge about ChREBP-mediated inter-organ crosstalk through secreted factors and its physiological implications. As the liver is considered a crucial endocrine organ, we will focus in this review on the role of ChREBP-regulated hepatokines. Lastly, we will discuss the involvement of ChREBP in the progression of metabolic pathologies, as well as how the impairment of ChREBP-dependent signaling factors contributes to the onset of such diseases.

## 1 Introduction

In order to maintain whole-body homeostasis, a complex network between organs has evolved in higher organisms, ensuring adaptation to external changes. Multidirectional interactions assure the absorption, sensing, storage and utilization of nutritional cues, boosting the efficiency of metabolic process. The communication between organs is orchestrated by the release of secreted messengers, including hormones, lipids and small molecules, which reach the target tissue transmitting the required message (1). Additionally, the nervous system contributes to the integration of all signals *via* direct tissue innervation (2). Disturbances of these coordinated interactions are associated to the development of metabolic diseases such as non-alcoholic fatty liver disease (NAFLD), type 2 diabetes (T2D) and obesity (3).

Originally known for its glucose-mediated control of glycolysis and lipogenesis, carbohydrate response element-binding protein (ChREBP) has emerged as an essential metabolic coordinator. ChREBP is the transcription factor mediating the response to carbohydrates. In the presence of glucose metabolites, glucose-response activation conserved element (GRACE) domain is stimulated, inducing ChREBP translocation to the nucleus. The reversal mechanism occurs through the activation of low-glucose inhibitory domain (LID), which in turn inhibits GRACE activity under low glucose concentrations. Both domains are enclosed in the Mondo conserved region (MCR), a highly evolutionarily conserved domain located in N-terminus of the Mondo family members (4). Of note, a new isoform of ChREBP named ChREBPβ has been described, which lacks the LID domain and is therefore constitutively active (5). ChREBP can undergo post-translational modifications such as acetylation and O-GlcNacylation, activating the efficiency in which ChREBP transcribes its target genes by increased binding affinities, stability and transcriptional activity under high glucose concentrations (6). In the nucleus, ChREBP forms a heterotetramer with max-like protein (MLX) and binds to the carbohydrate response element (ChoRE) present on the promoter of its target genes (4). In addition to the regulation of glycolytic (liver pyruvate kinase (*Lpk*)) and lipogenic genes (fatty acid synthase (*Fasn)*, stearoyl-CoA desaturase-1 (*Scd1)* and acetyl-CoA carboxylase 1 (*Acc1)* (7) *–* more details below), ChREBP controls the expression of a vast number of genes which exert tissue-specific functions. ChREBP expression is mostly abundant in liver and white/brown adipose tissues (8) but it is also expressed in pancreatic islets (9), small intestine (8) and to a lesser extent in kidney (8), adrenal glands (10) and brain (8). ChREBP modulates pancreatic insulin production and secretion through its control of antioxidant/mitochondrial biogenic programs (nuclear factor erythroid 2-related factor 2 (*Nrf2*)), as well as pro-oxidative/apoptotic pathways (thioredoxin interacting protein (*Txnip*)) (11, 12) and fructose absorption and metabolism (glucose transporter 2/5 (*Glut2/5*)), ketohexokinase (*Khk*), aldolase B (*AldoB*), triose phosphate isomerase (*TrioK*) and lactate dehydrogenase (*Ldh*) in the small intestine (13). In white adipose tissue (WAT), ChREBP is involved in adipocyte differentiation (peroxisome proliferator-activated receptor γ (*Pparγ*)) (14) and the production of palmitic-acid-hydroxy-stearic-acid (PAHSA), an insulin sensitiser lipid species (15). Finally, ChREBP regulates the hepatic production of bile acids (16) and hepatokines (17), both of them acting through endocrine signaling essential for homeostasis. Thus, ChREBP participates in many aspects of body physiology regulation *via* local and distant actions. The latter is driven by the release of secretory proteins through blood stream which allows inter-organ crosstalk (10, 17), the topic of the current review.

### 1.2 Role of ChREBP in liver metabolism

The liver mediates the conversion of excess dietary carbohydrates to fat through *de novo* lipogenesis (18). In the fed state, glucose is first converted into pyruvate (glycolysis) by the activation of several glycolytic enzymes, including liver pyruvate kinase (LPK). Pyruvate is then converted to acetyl-CoA and enters the tricarboxylic acid (TCA) cycle in the mitochondria for energy production. TCA cycle leads to an increase in citrate production, which serves as a substrate for *de novo* lipogenesis. Citrate is transported to cytosol and releases acetyl-CoA by ATP citrate lyase, activating lipogenic genes. The resulting acetyl-CoA is converted to malonyl-CoA, a key intermediary metabolite in fatty acid synthesis, by ACC1. Malonyl-CoA is a substrate of palmitate formation catalysed by fatty acid synthase (FASN). Finally, palmitate undergoes the desaturation and elongation reactions respectively by stearoyl-CoA desaturases (SCDs) and fatty acyl-CoA elongase (ELOVL) family members triggering fatty acid production, including stearic acid, palmitoleic acid and oleic acid (19). Regulation of lipogenic gene expression relies on the synergetic activity between ChREBP and sterol regulatory element-binding protein 1c (SREBP-1c) in response to glucose and insulin respectively, whereas the induction of LPK, a rate-limiting enzyme of glycolysis, is exclusively dependent on ChREBP (17). Global and liver-specific ChREBP deficient mice exhibit decreased glycolytic and lipogenic gene expression in liver (8, 20). ChREBP inhibition *via* RNA interference or genetic ablation prevents hepatic lipid accumulation in *ob/ob* mice through a specific decrease in lipogenic activity (21, 22). Moreover, ChREBP overexpression increases lipogenic gene expression leading to hepatic lipid accumulation (23). These models underline the direct involvement of ChREBP in *de novo* lipogenesis transcriptional control.

Once synthesized, hepatic triglycerides that are not stored as lipid droplets or metabolized through the β-oxidation pathway will be exported through the bloodstream in the form of very low-density lipoproteins (VLDL) to reach peripheral organs (18, 24). This process is controlled by hepatic microsomal triglyceride transfer protein (MTTP), by assembling and secreting apoliprotein B-containing lipoproteins. ChREBP was suggested as a potential VLDL regulator since putative loss-of-function ChREBP variants in humans lead to reduced plasma triglyceride levels (25). Mice globally lacking ChREBP and consuming a high fat/high carbohydrate diet show decreased hepatic *Mttp* expression and VLDL secretion rates, resulting in lower plasma triglyceride concentrations (26, 27). Nevertheless, no ChoRE could be identified on the *Mttp* promoter, suggesting that other transcription factors might be involved in transcriptional regulation of *Mttp* expression. In addition, ChREBP was recently identified as a direct transcriptional regulator of transmembrane 6 superfamily member 2 (TM6SF2), also involved in VLDL metabolism (28), reinforcing the importance of ChREBP in the control of lipoprotein secretion.

### 1.3 Potential effects of ChREBPβ in inter-organ communication

Over the past years, divergent results have highlighted the need to investigate the two ChREBP isoforms separately. The constitutively active isoform ChREBPβ is present in different tissues, owning tissue specific roles. First identified in WAT, ChREBPβ is expressed from an alternative promoter by ChREBPα, triggering a feed-forward regulation, where ChREBPβ is able to activate its own expression by binding to its promoter (5, 29). Herman and colleagues reported that adipose ChREBPβ is highly regulated in insulin resistant states implying a manner to predict insulin resistance (5). In agreement, deletion of mechanistic target of rapamycin complex 2 (mTORC2) subunit RICTOR in mature adipocytes decreases ChREBPβ expression, which reduces *de novo* lipogenesis in WAT, and impairs hepatic insulin sensitivity, emphasizing the role of ChREBPβ in the communication between WAT and liver (30). Although adipocyte ChREBPβ was reported to have insulin sensitizer properties, global *Chrebpβ* deficiency *via* CRISPR/Cas9-mediated gene editing showed mild alteration on ChREBP target genes (in WAT and liver), systemic insulin action and energy balance (31). Of note, DNA microarray analyses showed an impact of ChREBPβ deficiency on target genes expressed in brown adipose tissue (BAT) despite no apparent alteration in thermogenesis. Paradoxically, another study showed that BAT-specific overexpression of ChREBPβ inhibits BAT thermogenesis by downregulating uncoupling protein 1 (UCP1) and mitochondrial function, suggesting that BAT is a major site for ChREBPβ activity (32). Lastly, ChREBPβ is highly expressed in response to glucose in pancreatic β cells and its knockdown in both INS-1 cells and isolated rat islets decreased expression of ChREBP-dependent genes as well as glucose-stimulated proliferation. This indicates that ChREBPβ is the isoform that mediates the full proliferative effect of ChREBP (29). Altogether, these results reinforce the need to expand the current knowledge about the roles of the ChREBP different isoforms, as well as their specific roles in inter-organ crosstalk.

## 2 ChREBP-controlled secreted factors promote inter-organ dialogue

### 2.1 ChREBP regulates the expression of key hepatokines

**Figure 1:**
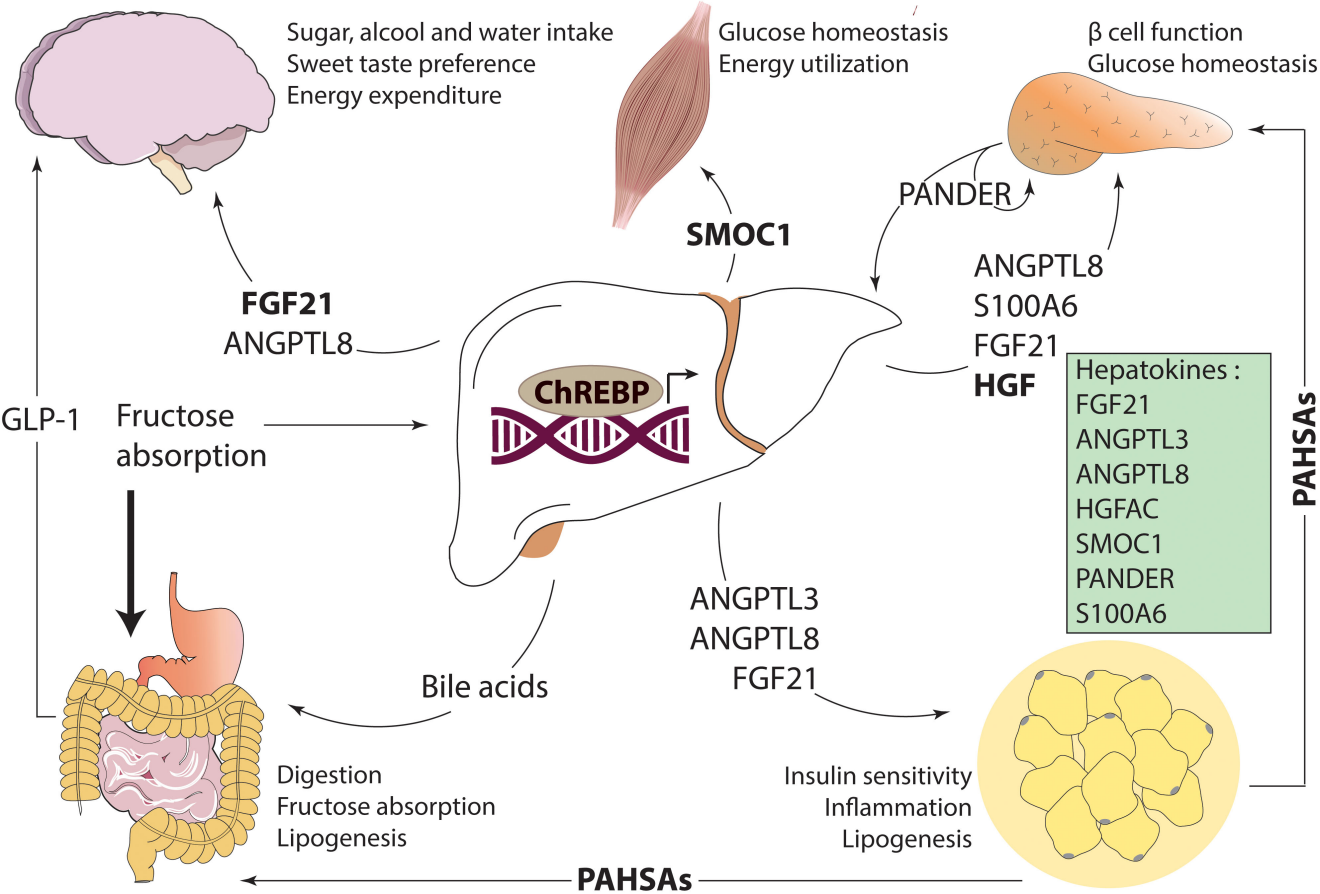
In health, ChREBP drives inter-organ communication through the regulation of different signaling factors in response to glucose and fructose. In the liver, ChREBP promotes the production of bile acids which are released in the intestine facilitating digestion and improving glucose homeostasis by GLP-1. ChREBP activity also triggers hepatokine secretion, such as FGF21, ANGPTL3, ANGPTL8, HGFAC, PANDER, SMOC1 and S100A6. These hormones reach their target tissues *via* the circulation and acts in specific tissues to regulate metabolic homeostasis. Finally, adipose ChREBP improves local and systemic insulin resistance by releasing PAHSAs.

Hepatokines are key actors of the modulation of metabolic adaptations through autocrine, paracrine, and endocrine signaling pathways important for controlling energy homeostasis (33) (**Figure 1**). Interestingly, several hepatokines have been described as direct ChREBP targets (4). The function of these ChREBP-dependent hepatokines is discussed below.

#### 2.1.1 Fibroblast growth factor 21

Secreted into the blood stream, FGF21 acts as an extracellular signal through a receptor complex that consists of a FGF receptor (FGFR) and a co-receptor β-Klotho. While its expression is more abundant in the liver, *Fgf21* has been reported in other organs, such as thymus, WAT, BAT, pancreas and heart, causing a direct effect *via* autocrine signaling or indirect impact *via* the central nervous system (34). FGF21 is linked to many beneficial effects in peripheral tissues driving the maintenance of energy homeostasis (35). In mice, *Fgf21* administration/overexpression is protective, preventing obesity in animals with diet-induced obesity, and improving hepatic triglyceride concentrations, cholesterol levels, energy expenditure and insulin sensitivity (36, 37). Similar results were observed in humans: the effects of LY2405319 (LY), a variant of FGF21 were tested in a randomized, placebo-controlled, double-blind trial in patients with obesity and T2D. LY treatment for 28 days significantly improved dyslipidemia, body weight and fasting insulin concentrations (38), indicating a potential therapeutic use of FGF21 for treating metabolic disorders (39).

FGF21 was first identified as a fasting hormone, enhancing lipolysis, β-oxidation and ketogenesis under the transcriptional control of peroxisome proliferator–activated receptor α (PPARα). However, recent studies also reveal that carbohydrate intake stimulates hepatic expression of *Fgf21* through ChREBP transcriptional activity (40, 41) (**Figure 1**). Genetic studies in human cohorts demonstrated a link between FGF21 and sugar preference. Single nucleotide polymorphisms at the *Fgf21* locus were associated with increased carbohydrate preference, but no relationship with ChREBP was reported (42, 43). Later studies revealed that liver ChREBP regulates sugar intake *via* FGF21 production which in turn decreases sweet taste preference (44, 45). The authors observed that *Fgf21* deficient mice have increased sucrose consumption while *Fgf21* overexpression or pharmacological treatment is associated with reduced intake of simple sugars and non-caloric sweeteners, but not of proteins or lipids. This effect was lost upon *β-Klotho* deletion in the hypothalamus, suggesting that this region of the brain is an important target site for FGF21 macronutrient intake regulation (44). Jensen-Cody et al. precisely described the action of FGF21 in ventromedial hypothalamus signaling to be dependent on glutamatergic neurons, uncovering the neuronal circuit involved in sugar intake and preference (46). Besides sugar intake, the ChREBP-FGF21 axis may also be involved in the control of other dietary constituents. *Fgf21* deficient mice showed increased alcohol intake while FGF21 administration/overexpression reduced alcohol preference (45). Additionally, ketogenic diet or alcohol ingestion significantly decreased water consumption in mice lacking *Fgf21* compared to control mice (47). Collectively, these data support the concept that ChREBP acts as a systemic modulator by controlling the liver-to-brain axis thereby generating a feedback loop involved in food behavior regulation (17).

#### 2.1.2 Angiopoietin-like protein 8

In the fed state, the VLDL uptake into peripheral tissues is facilitated by the action of lipoprotein lipase (LPL), which hydrolyses circulating triglycerides into free fatty acids for replenishing triglyceride reserves (18). The ChREBP-dependent hepatokine ANGPTL8 (also called lipasin, betatrophin or RIFL - refeeding induced in fat and liver) is a crucial player in this process, since it regulates triglyceride metabolism by affecting LPL activity (48). Stimulated by feeding, ANGPTL8 has been recognized as a direct ChREBP target due to the identification of a ChoRE on the *Angptl8* promoter ranging from -398 bp to -382 bp from the transcriptional binding site (49). Indeed, it has been shown that ChREBP binds to the *Angptl8* promoter in HepG2 cells (50). Additionally, mice treated with an adenovirus expressing a constitutively active form of ChREBP showed increased hepatic *Angptl8* expression levels (51)*. Angptl8* is also regulated by insulin through direct binding of liver X receptor α (Lxrα) (52), as well as LXRα-mediated expression of *Chrebp* and *Srebp1-c* (53, 54).

ANGPTL8 is expressed in liver and adipose tissue, and secreted into the circulation by the liver of mice and humans (55). Indeed, mice treated with liver-specific human *ANGPTL8* adenoviruses rapidly increased ANGPTL8 and triglyceride concentrations in plasma (55). ANGPTL8 is functional when it interacts with angiopoietin-like protein 3 (ANGPTL3) in the liver or angiopoietin-like protein 4 (ANGPTL4) in the adipose tissue. Due to their homology in protein structure, ANGPTL8 requires ANGPTL3 co-expression and complex formation to allow the maximal inhibition of LPL (56). Conversely, the ANGPTL4-8 complex impairs the ability of ANGPTL4 to repress LPL, favouring fatty acid uptake in adipocytes. Thus, ANGPTL8 functions as a metabolic switch in order to direct the energy from triglycerides in blood according to the metabolic needs (57). In addition to its role in lipid metabolism, ANGPTL8 exerts tissue-specific functions. In the hypothalamus, ANGPTL8 inhibits appetite *via* neuropeptide Y neurons (58). Since ChREBP regulates macronutrient intake through FGF21, inhibition of food intake by ANGPTL8 could represent another negative feedback loop dependent on ChREBP in order to maintain energy homeostasis (**Figure 1**). Moreover, ANGPTL8 seems to have an important role in adipogenesis. Ren et al. observed that during differentiation of mouse and human primary preadipocytes, the transcriptional levels of ANGPTL8 increased significantly with lipid droplet formation, along with changes in specific biomarkers of adipocyte differentiation (59). Mechanistic studies demonstrated that ANGPTL8 could promote adipocyte differentiation by inhibiting the Wnt/β-Catenin pathway and upregulating liver PPARγ expression (60), a ligand-activated nuclear receptor essential for adipocyte differentiation (61). Interestingly, ChREBP is also highly induced during differentiation of 3T3-L1 cells under high glucose concentrations. According to the authors, ChREBP activation induces PPARγ, by a mechanism dependent on the synthesis of a PPARγ regulatory lipid ligand (14). Thus, adipogenesis may be dependent on the ChREBP-ANGPTL8 axis.

#### 2.1.3 Angiopoietin-like protein 3

ANGPTL3 is exclusively produced in hepatocytes and circulates into the bloodstream. As previously mentioned, the ANGPTL3-ANGPTL8 complex interacts with LPL in peripheral tissues and inhibits its lipase activity reducing clearance of triglycerides (56). *Angptl3* is a direct target of LXRα (62), but a recent study also proposed a direct transcriptional regulation of *Angptl3* by ChREBP. Hepatic *Chrebp* overexpression decreases hepatic mRNA and protein expression of ANGPTL3, which was associated to a parallel reduction of plasma triglyceride concentrations (51). This contradicts the above mentioned report showing that lack of ChREBP decreases VLDL secretion rates, resulting in lower plasma triglycerides (26). These discrepancies suggest a double action of ChREBP in triglyceride level balance. ChREBP inhibition reduces *de novo* lipogenesis and *Mttp* expression, decreasing VLDL formation and consequently circulating triglycerides. Paradoxically, ChREBP activation increases hepatic lipid synthesis and LPL activity in WAT through decreased ANGPTL3, resulting in lower circulating triglyceride concentrations. This effect may represent a compensatory mechanism in order to maintain lipid homeostasis (51).

#### 2.1.4 Hepatocyte growth factor activator

HGFAC is a liver-secreted protein that activates hepatocyte growth factor (HGF), which regulates many biological effects including embryonic development, cell migration, epithelial morphogenesis, inflammation, apoptosis and proliferation in different tissues *via* its specific receptor tyrosine kinase, MET (63, 64). While the liver is the main site of *Hgfac* expression, it is also expressed at lower levels in other tissues such as the intestine and possibly the pancreatic islets (65, 66). Sargsyan et al. have recently demonstrated that HGFAC plays a role in systemic metabolism through ChREBP activity (66) (**Figure 1**). Liver-specific ChREBP knockout mice exhibit decreased *Hgfac* expression compared to controls when consuming a high fructose diet. Genotype-Tissue Expression Biobank analysis supports the hypothesis that ChREBP-mediated regulation of *HGFAC* is also conserved in humans, since well-validated ChREBP target genes showed strong correlation with the expression of HGFAC (66). Interestingly, *Hgfac* global knockout mice exhibit reduced hepatic and increased circulating triglyceride concentrations respectively, with a parallel glucose intolerance and insulin resistance, suggesting that HGFAC plays a key role in the regulation of lipid clearance and glucose homeostasis. The metabolic defects observed in *Hgfac* global knockout mice can be mechanistically explained by the downregulation of *Pparγ* and its target genes (66, 67). Since HGF presents pleiotropic biological activities, ChREBP-mediated HGFAC secretion might be involved in other adaptive responses. Mice lacking pancreatic HGF receptor showed decreased β cells mass and impaired glucose tolerance and insulin secretion (68). In agreement, inhibition of *Chrebp* in pancreatic β cells leads to decreased glucose-stimulated proliferation in rat and human cells. (69). Altogether, these results suggest that ChREBP mediates an adaptive response to glucose overconsumption *via* activation of the HGFAC-HGF axis to preserve glucose and lipid homeostasis.

#### 2.1.5 Pancreatic-derived factor

PANDER, also known as family with sequence similarity 3 member B (FAM3B), was first described as a β cell cytokine facilitating insulin secretion under physiological conditions (70). However, recent work revealed another biological role for PANDER in the regulation of glycemia *via* an interaction between liver and pancreas. PANDER is constitutively expressed and secreted by the liver in mice and humans, and increased under diabetic condition (71). While previous studies reported that the glucose-responsiveness of PANDER in pancreatic β cells is due, in part, to the pancreas-specific transcription factor Pancreatic Duodenal Homeobox-1 (PDX-1) (72), Ratliff et al. demonstrated that, in the liver, PANDER is stimulated by glucose through ChREBP, which directly binds to the *Pander* promoter (73). Liver-specific *Pander* overexpression leads to hepatic insulin resistance, and increased gluconeogenesis and lipogenesis, resulting in hepatic steatosis onset in mice. This is associated with increased SREBP-1c and FAS protein levels in mouse livers (71), suggesting that, besides its direct transcriptional effect on *de novo* lipogenesis, ChREBP also regulates hepatic lipid deposition through PANDER upregulation (**Figure 1**). In addition to its local hepatic function, PANDER also impairs the production of glucagon-like peptide 1 (GLP-1), an incretin critical for glucose-dependent insulin secretion, in intestinal L cells *in vivo* and *in vitro (* 74). Herein, intestine might be a PANDER target tissue revealing another way of regulation of glucose and lipid metabolism *via* inter-organ communication (74).

#### 2.1.6 SPARC-related modular calcium-binding protein 1

Originally isolated from bone, SMOC1 is expressed in several tissues, including liver, heart, adipose tissues and skeletal muscle (75) with secretion coming exclusively from the liver. Studies have shown its involvement in embryogenesis, endothelial cell proliferation, angiogenesis, cell adhesion, and recently in glucose metabolism (76) (**Figure 1**). Due to the identification of two ChoRE consensus sequences within its promoter, *Smoc1* was identified as a target gene of ChREBP. This was confirmed by ChREBP knockdown *via* small interfering RNA *in vitro*, which blunted *Smoc1* expression in response to high glucose concentration (77). In mice, S*moc1* silencing is associated to impairment in glycemic control as well as insulin resistance. These defects are significantly improved by SMOC1 administration in obese and insulin resistant mice fed with a high fat diet, suggesting a potential therapeutic effect. The beneficial glycemic effect of SMOC1, acting through an orphan receptor, was attributed to decreased gluconeogenesis *via* the inhibition of adenosine 3′,5′-cyclic monophosphate (cAMP), decreasing cAMP response element–binding protein (CREB) signaling in the liver (77). This effect leads to suppression of hepatic glucose output as well as increased glucose uptake into mixed gastrocnemius skeletal muscle (77). In humans, SMOC1 plasma levels are decreased in insulin-resistant subjects, but no correlation was seen between SMOC1 plasma levels and liver lipid content (77). Besides its role in glucose metabolism, the ChREBP-SMOC1 axis could be involved in osteoblast differentiation. Indeed, Smoc1 is under transcriptional regulation of runt-related transcription factor 2 (RUNX2) (78), a transcription factor essential for bone formation and osteoblastogenesis which was identified as being dependent on ChREBP activity (79).

#### 2.1.7 Calcium-binding protein S100A6

Also known as calcyclin, S100A6 belongs to the S100 family of Ca^2+^–binding proteins. Identified in rodent fibroblasts (80), S100A6 was first characterized for its role in cell-cycle progression. However, its function was extended to the regulation of other cellular functions, such as proliferation, apoptosis, cytoskeleton dynamics, and the cellular response to stress factors (81). S100A6 is widely expressed with high expression in fibroblasts, epithelial cells, and to lesser extent in the brain, muscle, heart and liver (82). Elevated S100A6 expression correlates to T2D and pancreatic cancer (83). Exploring the pathophysiological relevance of S100A6, a recent study reported an association between increased S100A6 levels and NAFLD in both humans and mice (84). Mechanistic studies in HepG2 cells revealed the presence of a ChREBP binding site on the *S100a6* promoter, suggesting a direct control of S100A6 by ChREBP in response to glucose. The expression of the *S100A6* gene can also be activated by other transcription factors, such as upstream transcription factor (USF) (85), nuclear transcription factor kappa B (NFκB) (86), and NRF2 (87), in response to stimuli other than glucose (**Figure 1**). In mice, liver ChREBP-mediated upregulation of hepatic and serum S100A6 impairs insulin secretion from pancreatic β cells *via* the interaction with the receptor for advanced glycation end products (RAGE). S100A6 caused alterations in the synthesis of intracellular cAMP and inhibition of mitochondrial ATP production in β cells. Knockdown of S100A6 was sufficient to improve insulin secretion in β cells from mice overexpressing hepatic ChREBP (*via* adenovirus infection) (84). These results reveal a new axis between liver and pancreas, in which ChREBP affects β cell insulin secretory function.

### 2.2 Role of ChREBP in the regulation of bile acid metabolism

Bile acids are produced from cholesterol by hepatocytes and stored in the gallbladder in the fasted state. In response to a meal, bile acids migrate to the intestinal lumen where they facilitate digestion and absorption of dietary lipids and control gut microbial overgrowth (88). The mechanism behind these regulations depends on a negative feedback by bile acids themselves, process facilitated by the nuclear receptors pregnane X receptor (PXR) and farnesoid X receptor (FXR). Evidence shows that FXR is also important for glucose and lipid homeostasis *via* its direct interaction with ChREBP, FXR acting as a repressor on the ChoRE of glycolytic genes (16). In presence of high glucose concentrations, ChREBP forms a complex with hepatocyte nuclear factor 4α (HNF4α) and the transcription coactivators p300 and CBP on the ChoRE of ChREBP target gene promoters. In response to the concomitant presence of bile acids and glucose, FXR is activated leading to the recruitment of the co-repressor SMRT and the consequent release of CBP, p300 and ChREBP, a mechanism which represses glycolytic and lipogenic gene expression (16). Both ChREBP and FXR activity can be enhanced by O-GlcNAcylation, a post-translational modification that depends on glucose suggesting another way of interaction between both transcription factors (89). ChREBP and FXR interplay is linked to GLP-1 secretion by the intestine (**Figure 1**). Using murine intestinal GLUTag L cell line, Trabelsi et al. demonstrated that *Chrebp* knockdown through a RNA interference strategy prevents the glucose-induced induction of GLP-1 levels. While FXR activation by its agonist GW4064 inhibits *Glp-1* expression, this effect is lost when ChREBP was silenced (90), highlighting the importance of ChREBP in this process. In addition of its interaction with FXR, ChREBP is also implicated in the control of bile acid composition. Indeed, when activated, ChREBP induces the expression of hepatic sterol 12α-hydroxylase (CyP8b1 - the rate-controlling enzyme of the bile acid pathway) increasing the relative abundance of cholic-acid-derived bile acids. This change in composition alters the hydrophobicity of the bile acid pool, leading to a greater intestinal lipid solubilization and uptake (91).

### 2.3 Adipose ChREBP regulates novel lipid species

A novel class of mammalian lipids referred as fatty acids ester of hydroxy fatty acids (FAHFAs) has been recently identified (15). Characterized by a branched ester linkage between a fatty acid and a hydroxy-fatty acid, FAHFAs (especially PAHSAs) have been associated to beneficial effects on insulin and glucose tolerance (**Figure 1**). FAHFAs are found in BAT, subcutaneous and perigonadal WAT, liver, with lower extent in heart and gastrocnemius muscle of mice (15). In humans, concentrations of FAHFAs were detected in serum, breast milk, meconium, and adipose tissue (92). PAHSA levels in serum are positively correlated with insulin sensitivity while their levels in breast milk negatively correlate with obesity in nursing mothers (15, 93). Decreased levels of PAHSAs were also observed in mice with high-fat diet-induced insulin resistance (15). These effects occur in a ChREBP-regulated manner since ChREBP knockout mice exhibit diminished PAHSA serum concentrations (15). Mice with a deletion of ChREBP in adipose tissue (ChREBP^AdipoKO^) also exhibit low circulating PAHSA levels, implying that systemic production of PAHSAs depends on ChREBP expression in adipocytes. Interestingly, a recent paper describes a novel secreted adipokine named WNT1-inducible signaling pathway protein 2 (WISP2/CCN5), which enhances adipose precursor cell proliferation (94). Mice overexpressing WISP2 in WAT showed increased adipose lipogenic markers such as *Chrebp*, which correlated to increased PAHSA levels and improved insulin sensitivity (94). In this context, WISP2 may modulate PAHSA concentrations through its control of ChREBP. While the exact mechanism(s) by which ChREBP regulates PAHSA production has not been identified, ChREBP by regulating the lipogenic pathway, could be also directly implicated in the production and/or degradation of these lipids and thereby modulate insulin sensitivity.

Interestingly, 9-PAHSA supplementation fully rescued the phenotype of insulin resistance and the defect in glucose transport of ChREBP^AdipoKO^ mice (95). This is mediated through direct effects on insulin secretion or through a mechanism involving PAHSA-mediated secretion of incretins by intestinal L-cells. Indeed, it was reported that acute oral administration of either 5- or 9- PAHSA isomer improves glucose tolerance and increases insulin and GLP-1 secretion (15). In contrast, blockage of GLP-1 receptor through the use of the antagonist exendin 9-39 in mice reversed the beneficial effects of PAHSAs on glucose tolerance (96). The fact that ChREBP also controls the expression of the preproglucagon gene in GLP-1 producing cells, suggests a role for ChREBP in the incretin response (90). Moreover, PAHSAs act in the endocrine pancreas by increasing the activity of the G-protein coupled receptor 40 (GPR40), which triggers insulin secretion in β cells (96). In turn, increased insulin action stimulates glucose uptake in muscle and heart, and suppresses endogenous glucose production in liver and lipolysis in WAT (97). These studies highlight the role of ChREBP in regulating the production of PAHSAs, beneficial lipids which enhance insulin sensitivity through different mechanisms involving inter-organ communication.

## 3 Pathological implications of ChREBP

**Figure 2:**
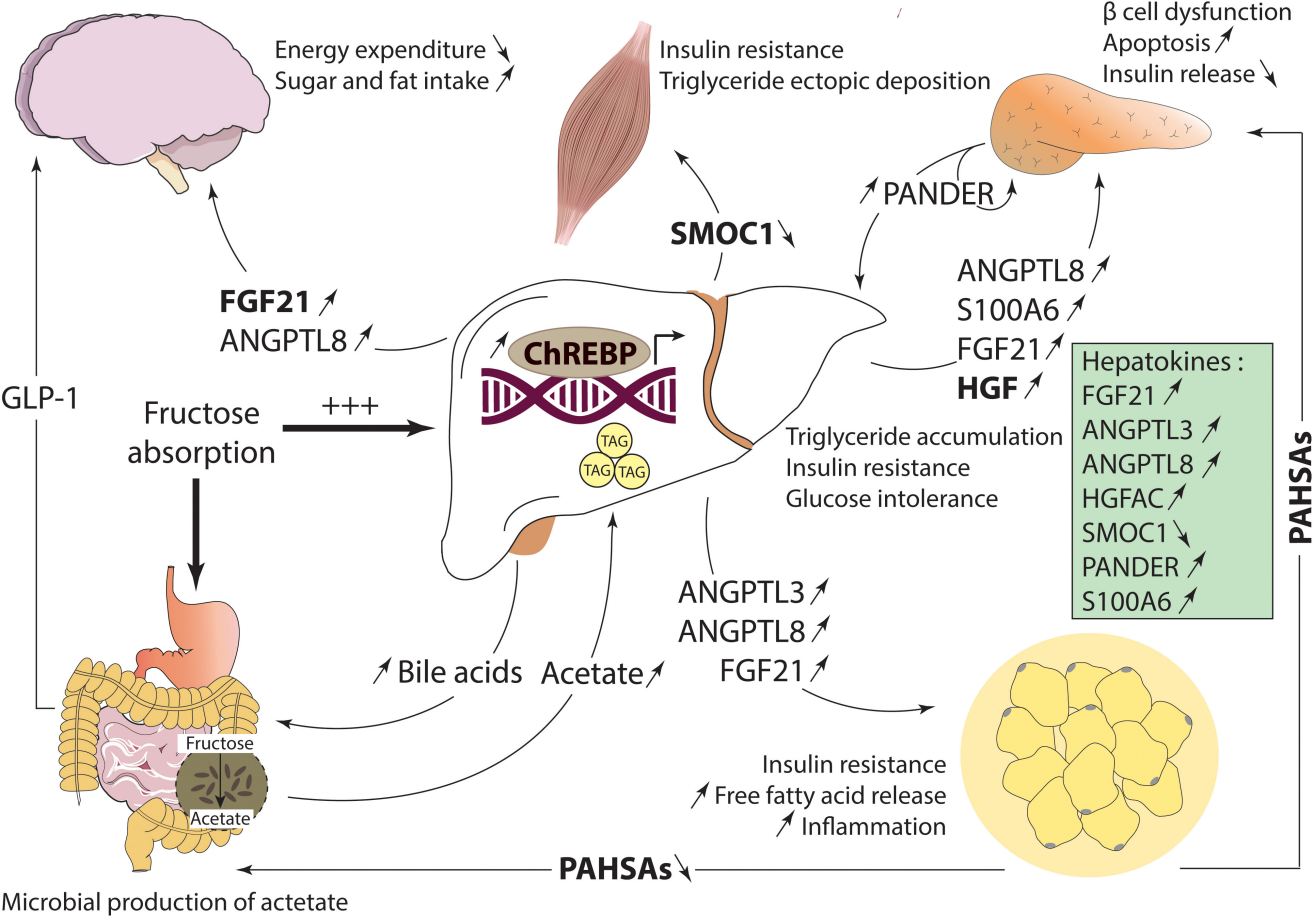
In disease, signaling factors levels are dysregulated due to altered activity of ChREBP. In the liver, ChREBP contributes to hepatic steatosis through direct stimulation of the lipogenic pathway. This is more notable in the case of fructose overconsumption, which accelerates the process *via* direct metabolization in the liver and acetate formation, a lipogenic substrate, by the microbiota in the intestine. At the periphery, ChREBP modulates the alteration in bile acids levels and composition, impairing dietary lipids absorption and GLP-1 secretion. Changes in ChREBP-dependent hepatokines levels are strongly associated to hepatic and systemic insulin resistance. Finally, in adipocytes, PAHSA secretion is decreased due to decreased ChREBP activity, which contributes to the insulin resistant state and disease development.

Excessive energy intake combined with a sedentary lifestyle imbalance the body homeostasis, causing an increase in the incidence of metabolic diseases, such as NAFLD, T2D and obesity (98). NAFLD is currently the most common liver dysfunction affecting between 14% and 34% of the general population, with higher rates among overweight individuals (70%) (99). Governed by alterations in hepatic and extra-hepatic signals, NAFLD is characterized by lipid accumulation in the liver resulting from disturbances of glucose and lipid metabolism (100). *De novo* lipogenesis is a major contributor of the disease, mostly due to an elevated hepatic ChREBP activity (21). Indeed, *Chrebp* expression is increased in liver of *ob/ob* mice with hepatic steatosis (21) and in human liver from patients developing simple hepatic steatosis and non-alcoholic steatohepatitis (23, 101). Interestingly, fructose has been closely associated to NAFLD, due to its rapid metabolization in the liver and its ability to strongly stimulate lipogenic programs through ChREBP (100). It has been reported that ChREBP activity is increased in liver of mice fed a high-fructose diet (102). In contrast, ChREBP inactivation, driven by inhibition of KHK which is essential for fructose metabolization, reverses fructose mediated hyperinsulinemia, hypertriglyceridemia and hepatic steatosis (103). ChREBP is involved in the direct fructose metabolization in the liver and in the indirect metabolization *via* acetate, a pathway dependent on the communication between intestine and liver. High fructose concentrations overwhelm the absorptive capacity of the intestine, resulting in fructose displacement into colonic microbiota (104). By the action of gut microbes, fructose is converted into acetate that is further transported *via* the portal vein into the liver. Once in the liver, acetate is used by the lipogenic pathway through its conversion to acetyl-CoA by acetyl-CoA synthetase 2 (ACSS2), an enzyme transcriptionally controlled by ChREBP (105), reinforcing the role of ChREBP in the spectrum of NAFLD development (**Figure 2**).

Worth mentioning is that high fructose consumption strongly stimulates ChREBPβ, rather than ChREBPα, which is accompanied by increased expression of glycolytic and lipogenic genes in the liver of mice (106). ChREBPβ activation drives lipogenesis and expression of patatin-like phospholipase domain-containing 3 (PNPLA3) a lipase that mobilizes stored triglycerides for VLDL secretion, increasing the flux of fatty acids to the skeletal muscle, thus resulting in insulin resistance in this tissue (107). ChREBPβ is also involved in the physiopathology of other tissues. In the kidney, mice fed high-fructose diet exhibit increased ChREBPβ and *de novo* lipogenesis gene expression, as well as increased triglyceride content (108). Finally, although essential for adaptive β cell expansion in physiological levels, elevated ChREBPβ expression stimulated by prolonged hyperglycemia leads to loss of β cell mass and identity, apoptosis, and diabetes, while its depletion protects pancreatic β cells from glucolipotoxicity (109).

In addition to increased lipid synthesis, disturbances in lipid secretion also drive hepatic steatosis. The VLDL secretion rate appears to depend, not only on the availability of hepatic triglycerides, but also on the overall capacity for VLDL assembly. Indeed, liver specific deletion of *Mttp* or *Tm6sf2*, two genes regulated by ChREBP, impairs VLDL secretion, promoting hepatic steatosis and fibrosis (110, 111). In addition, MTTP human polymorphisms are associated with reduced plasma over liver lipids and increased the risk of NAFLD development (112). Nevertheless, the secretion rate of VLDL-triglyceride is often increased in patients with NAFLD. This may be driven by decreased rates of lipid absorption due to altered levels and composition of bile acids (**Figure 2**). Changes in bile acid composition were associated to decreased FXR signaling contributing to the development of NASH in mice (113). In line, FXR activation reduced fatty acid synthesis through a bile-acid-dependent mechanism, protecting high-fat-fed mice against NAFLD (114). These results reinforce the critical role of the FXR-ChREBP interaction in the development of NAFLD *via* a gut-liver axis.

Most of the NAFLD patients are insulin resistant, rendering this disorder a strong risk factor for T2D development (115). It was reported that ChREBP could have a beneficial role on insulin sensitivity independently of hepatic steatosis. Indeed, liver and global deletion of ChREBP lead to glucose intolerance and insulin resistance in mice (8, 20), while liver-specific ChREBP overexpression in mice fed a high-fat diet ameliorates insulin signaling despite increased hepatic lipid accumulation (23). One explanation, as discussed above, is that ChREBP-dependent secretion of beneficial hepatokines could influence metabolic homeostasis through autocrine and/or paracrine signals (**Figure 2**). Alterations in hepatokine homeostasis strongly contribute to the development and the worsening of metabolic diseases, reinforcing their promising value as pathological biomarkers and/or pharmacological interventions (33). Of special interest is the hepatokine FGF21 which protects from hepatic steatosis and improves insulin sensitivity in mice and humans when administrated (36, 39). Lastly, adipose ChREBP by contributing to the release of PAHSAs may exert tissue-specific protective effects. Indeed, as mentioned, these lipids strongly correlate with insulin sensitivity (15). Administration of 5- and 9-PAHSA, two PAHSA isomers, improves glucose tolerance and insulin sensitivity in mice by suppressing endogenous glucose production inhibiting lipolysis and stimulating glucose uptake in glycolytic muscle and heart in high fat diet fed mice (97) (**Figure 2**). Altogether, acting in different tissues and regulating several inter-organ *dialogues*, ChREBP appears as a key metabolic regulator of energy homeostasis.

## 4 Conclusions and further perspectives

Primarily known for its transcriptional role on glycolytic and lipogenic genes, ChREBP has emerged as a critical regulator of a broad range of processes across the body, with physiological and pathological implications. This review integrated the recent advances on ChREBP mediated inter-organ communication through the control of peptide and lipid production/secretion (**Figure 1**). The liver, as a central endocrine organ, regulates the production of bile acids and hepatokines through ChREBP. Although it is clear that ChREBP influences the expression of the hepatokines mentioned in this review, the identity of many others remains unknown. This opens doors to new discoveries of ChREBP-controlled hepatokines, as well as their receptors and signaling pathways, aiming for the improvement of diagnosis (as biomarkers) and therapeutic treatment for metabolic diseases. Extra-hepatic ChREBP is also involved in the preservation of metabolic homeostasis. Adipose ChREBP promotes the synthesis of beneficial lipids species named PAHSAs. Moreover, evidence implies that local ChREBP activity in the brain may play an integrating role given the effect of FGF21 and ANGLPT8 on satiety control and the neuroprotective role of bile acids (116) and PAHSAs (117), although its direct contribution remains to be addressed. Further analysis of the role of ChREBP in other tissues such as BAT, kidney, gut, and adrenal glands is required and mouse models with tissue specific deletions for ChREBP may facilitate the understanding of their function in inter-organ networks. While coordination of multidirectional interactions between tissues ensures metabolic homeostasis, its dysregulation is associated to NAFLD and T2D (**Figure 2**). In this regard, the role of ChREBP in fructose metabolism should be further explored given its ability to strongly activate lipogenic program, leading to hepatic fat accumulation and systemic insulin resistance. ChREBPβ, rather than ChREBPα, is strongly stimulated by fructose, fact that reinforces the need to study both isoforms separately in physiology and pathophysiology.

## Author contributions

All authors listed have made a substantial contribution to the work and approved it for publication. TC and CP wrote the manuscript. MR designed the figures. LP, MR, FB provided critical reading of the manuscript.

## Glossary

**Table:**

ACC1	acetyl-CoA carboxylase 1
ACSS2	acetyl-CoA synthetase 2
ALDOB	aldolase B
ANGPTL3	angiopoietin-like protein 3
ANGPTL4	angiopoietin-like protein 4
ANGPTL8	angiopoietin-like protein 8
BAT	brown adipose tissue
cAMP	adenosine 3'-5'-cyclic monophosphate
ChoRE	carbohydrate response element
ChREBP	carbohydrate response element binding protein
CREB	cAMP response element–binding protein
CyP8B1	hepatic sterol 12α-hydroxylase
ELOVL	fatty acyl-CoA elongase
FAHFAs	fatty acids ester of hydroxy fatty acids
FAM3B	family with sequence similarity 3 member B
FASN	fatty acid synthase
FGF21	fibroblast growth factor 21
FGFR	fgf receptor
FXR	farnesoid X receptor
GLP-1	glucagon-like peptide 1
GLUT2/5	glucose transporter 2/5
GPR40	G-protein coupled receptor 40
GRACE	glucose-response activation conserved element
HGF	hepatocyte growth factor
HGFAC	hepatocyte growth factor activator
HNF4α	hepatocyte nuclear factor 4α
KHK	ketohexokinase
LDH	lactate dehydrogenase
LID	low-glucose inhibitory domain
LPK	liver pyruvate kinase
LPL	lipoprotein lipase
LXRα	liver X receptor α
LY	LY2405329
MCR	Mondo conserved region
MLX	max-like protein
mTORC2	mechanistic target of rapamycin complex 2
MTTP	microsomal triglyceride transfer protein
NAFLD	non-alcoholic fatty liver disease
NF-κB	nuclear transcription factor- kappa B
NRF2	nuclear factor erythroid 2-related factor 2
PAHSA	palmitic-acid-hydroxy-stearic-acid
PANDER	pancreatic-derived factor
PNPLA3	patatin-like phospholipase domain-containing 3
PDX-1	pancreatic duodenal homeobox-1
PPARα	peroxisome proliferator–activated receptor α
PPARγ	peroxisome proliferator-activated receptor γ
PXR	pregnane X receptor
RAGE	receptor for advanced glycation end products
RUNX2	runt-related transcription factor 2
SCD1	stearoyl-CoA desaturase-1
SCDs	stearoyl-CoA desaturases
SMOC1	SPARC-related modular calcium-binding protein 1
SREBP-1c	sterol regulatory element-binding protein 1c
S100A6	calcium-binding protein S100A6
T2D	type 2 diabetes
TCA	tricarboxylic acid
TM6SF2	transmembrane 6 superfamily member 2
TRIOK	triose phosphate isomerase
TxNIP	thioredoxin interacting protein
UCP1	uncoupling protein 1
USF	upstream transcription factor
VLDL	very low-density lipoproteins
WAT	white adipose tissue
WISP2/CCN5	WINT1-inducible signaling pathway protein 2
